# Hypothalamic microRNAs flip the switch for fertility

**DOI:** 10.18632/oncotarget.14646

**Published:** 2017-01-13

**Authors:** Andrea Messina, Vincent Prevot

**Affiliations:** Laboratory of Development and Plasticity of the Neuroendocrine Brain, Inserm U1172, University of Lille, School of Medicine, Lille, France; Service d’Endocrinologie, diabétologieet métabolisme, Centre Hospitalier Universitaire Vaudois, Lausanne, Switzerland

**Keywords:** microRNA, GnRH, puberty, fertility, gene network

Reproduction, in mammals, is finely regulated by the hypothalamic-pituitary-gonadal axis (HPG) and driven, by a complex neural network within the hypothalamus that converges onto GnRH-producing neurons, the master regulators of gonadotropin secretion [1]. The correct development of the GnRH system as well as a finely programmed postnatal increase in GnRH production and secretion are essential for sexual maturation and puberty onset [1]. However, despite the identification of several factors known to influence GnRH production and release, the molecular mechanisms controlling the postnatal increase in GnRH expression and its timing have remained a mystery until now. We have recently made a significant advance in understanding these processes by uncovering a specific set of microRNAs, embedded in the genetic network controlling GnRH transcription, that drive the infantile switch from the repression to the induction of GnRH expression [2].

MicroRNAs are small, non-coding RNAs that fine-tune gene expression at the post-transcriptional level. Considered major players in epigenetics, microRNAs, together with transcription factors, form integrated gene regulatory networks that can control complex cellular processes with extreme temporal precision [3], making them ideal candidates for the molecular control of the timing of puberty. Converging evidence supports a role for microRNAs in mouse gonadal [4] and pituitary function [5], while a recent human genome-wide association study has highlighted a link between age at puberty and a genetic locus controlling the expression of a specific microRNA family [6]. However there has been no direct evidence until now of the functional implication of microRNAs in the hypothalamic control of puberty, nor of the mechanisms that could subtend this process.

**Figure 1:**
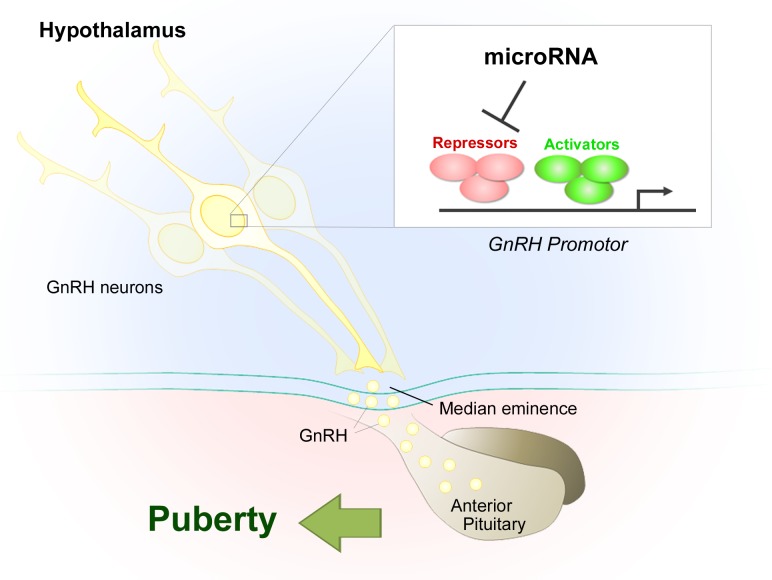
MicroRNA-gene network controlling GnRH transcription: the timely switch in GnRH expression from repression to induction leads to puberty onset

Our results show that, during an infantile “critical period” lasting just a few days, a dramatic switch in the microRNA expression pattern of GnRH neurons inverts the balance between inductive and repressive signals, triggering increased hypothalamic GnRH expression and controlling the crucial transition from the early infantile phase, when its levels are low, to the GnRH-fuelled run-up to puberty (Figure 1) [2]. This infantile “critical period” corresponds to a centrally-driven gonad-independent activation of the HPG axis and the resulting surge in gonadotropin levels (on postnatal days 10-16 in mice). This phenomenon, known as “mini-puberty” in humans, is the first of three activational periods that prime the HPG axis for puberty and adult fertility, setting in motion, for example, the growth of the first wave of ovarian follicles set to ovulate at puberty in females and the development of the testes in males [7].

Specifically, we have shown that during this critical period, a switch in the expression of several microRNA species in the hypothalamus in turn flips a switch in a multilayered array of GnRH gene activators and repressors, permitting the sustained increase of the neurohormone required for subsequent sexual maturation [2]. Two microRNA species act as the linchpins of this process: the miR-200/429 family, which is not only upregulated during the critical period but selectively enhanced in GnRH neurons, and miR-155, which appears to act on other hypothalamic cell types as well, and mediates, for example, the effects of a release of nitric oxide known to occur concomitantly upstream of GnRH neurons. We have also pinpointed several genes targeted directly or indirectly by miR-155 and miR-200/429, whose repression (e.g. Cebpb and Zeb1) or activation (e.g. Pou2f1 and Meis1, which are both targets for Zeb1) is required for the normal increase of GnRH expression from “mini-puberty” onwards. Selectively blocking miR- 200/429 binding to the 3′UTR of the Zeb1 transcript in the hypothalamus during the infantile period (between P7 and weaning) suppresses GnRH promoter activity at the cellular level and leads to abnormalities in the onset of puberty. Interestingly, the microRNA-gene micronetworks that we have identified as sustaining the postnatal increase in GnRH expression also appear to be at work during adulthood, since the blockade of miR-200/429/Zeb1 binding in the hypothalamus impacts estrous cyclicity in adult female mice.

These results raise the intriguing possibility that the microRNA-dependent epigenetic regulation of GnRH secretion could underlie the pathophysiology of human congenital hypogonadotropic hypogonadism (CHH) when no mutations are found in known CHH genes [8]. Exploring the putative contribution of mutations in these microRNA-gene networks could thus hold therapeutic potential for disorders of puberty and fertility of hypothalamic origin (e.g. by using small molecules that interfere with or restore microRNA action on target genes).

While the identification of this unprecedented microRNA-based genetic network controlling GnRH transcription represents a clear advance in our understanding of the molecular basis of puberty, it raises new questions and challenges. What triggers this developmental switch in microRNA expression in GnRH neurons after birth? What roles do hypothalamic wiring; hormones and the environment play in this process? And finally, to what extent can pharmacological interventions modulate this process, and what are their limitations?
